# Urine amylase level after Whipple resection might be a predictive factor of post-operative complications

**DOI:** 10.17305/bjbms.2022.7356

**Published:** 2022-06-03

**Authors:** Farid Ljuca, Amir Tursunović, Kenana Ljuca, Zijah Rifatbegović, Mirha Agić

**Affiliations:** 1 Department of Physiology, School of Medicine, University of Tuzla, Tuzla, Bosnia and Herzegovina; 2 Department of Surgery, University Clinical Center Tuzla, Tuzla, Bosnia and Herzegovina; 3 School of Medicine, University of Tuzla, Tuzla, Bosnia and Herzegovina

**Keywords:** Urine amylase level, Whipple resection, post-operative complications, post-operative pancreatic fistula

## Abstract

The association between urine amylase levels and the development of post-operative complications after Whipple resection is still unknown. The aim of this study was to determine the prognostic value of urine amylase levels for post-operative complications in patients who underwent Whipple resection. In this retrospective-prospective cohort study we analyzed amylase levels in urine, serum, and drains in 52 patients who underwent Whipple resection preoperatively and on Post-operative Day 1 (POD1) after the intervention. Patients were followed up for 3 months to assess their predictive value for post-operative complications. In patients with complications, urine amylase levels were significantly higher on POD1 than before resection (198.89 ± 28.41 vs. 53.70 ± 7.44, *p*=0.000). Considering the sensitivity and specificity of the urine amylase level on POD1, an area under the ROC curve of 0.918 was obtained (*p*<0.001, 95% Confidence interval [CI]: 0.894-0.942). Patients with urine amylase levels ≥140.00 U/L had significantly higher risks of post-operative pancreatic fistula (POPF) grade C (definition of POPF done according to the ISGP) (RR:20.26; 95% CI: 1.18-347.07; *p*=0.038), readmission to hospital (RR: 6.61; 95% CI: 1.53-28.58; *p*=0.011), reoperation (RR: 5.67; 95% CI: 1.27-25.27; *p*=0.023), and mortality (RR:17.00; 95% CI: 2.33-123.80; *p*=0.005) than patients with urine amylase levels <140.00 U/L. Urine amylase levels on POD1 displayed strong and significant positive correlations with serum amylase levels (*r*=0.92, *p*=0.001) and amylase levels in drains (*r*=0.86, *p*=0.002). We can conclude that urine amylase levels on POD1 have good prognostic value for post-operative complications after Whipple resection and might be used as an additional predictive risk factor.

## INTRODUCTION

In treating of benign and malignant tumors, Whipple resection is a well-standardized procedure. Despite continuous improvements of surgical techniques and perioperative treatment over the last decades, after Whipple resection, the incidence of mortality and post-operative complications was still high [1,2]. The most frequent and the most severe post-operative complication after Whipple resection are post-operative pancreatic fistula (POPF). The most appropriate definition and grading of POPF established by the International Study Group on Pancreatic Fistula (ISGPF) [3]. The incidence of POPF is between 11 and 16% of cases, and it is usually treated conservatively [4]. POPF is often associated with other complications such as hemorrhage, abscess, sepsis, and abdominal collections which require extended hospitalization or readmission to the hospital and reoperation [5-7]. Whipple resection and its post-operative complications may cause dysfunction of the exocrine pancreas. Therefore, the markers for pancreatic function may be used as predictors of the development of post-operative complications after Whipple resection. Several studies [8-11] have shown an association between amylase levels in drains and serum with post-operative complications after pancreaticoduodenectomy.

The aim of this study was to determine the prognostic value of urine amylase levels for post-operative complications in patients who underwent Whipple resection.

## MATERIALS AND METHODS

In this retrospective-prospective cohort study 52 patients who underwent Whipple resection at the Clinic of Surgery, University Clinical Center Tuzla, Bosnia and Herzegovina were enrolled from September 1, 2016 to April 1, 2021. In all patients who underwent Whipple resection preoperatively and on POD1 after the intervention, we analyzed the markers for the exocrine pancreas function (amylase levels in drains, serum, and urine). The markers in drains, serum, and urine were measured using ELISA test. For measurement of amylase, 10 mL of liquid (serum, urine, and drains) were used.

Including criteria for the enrollment of the patients in the study were as follows: (a) malignant tumors of the pancreas and the papilla Vateri proven preoperatively by histopathological analysis; (b) malignant tumors of the pancreas without metastasis; and (c) patients treated only by Whipple resection.

Excluding criteria for this study were as follows: (a) malignant tumors of the pancreas with metastasis; (b) benign tumors of the pancreas; and (c) patients treated by some kind of palliative procedure.

The patients underwent a follow-up for 3 months after Whipple resection. Over that time, all developed complications (POPF, delayed gastric emptying, abdominal collections, enteric fistula, readmission to hospital, post-operative bleeding, reoperation, and mortality) were recorded.

Complications were classified according to the definition by Clavien and Dindo [12]. POPF was classified as Grades A, B, and C according to the definition established by the ISGPF [3].

All patients were administered pre-operative antibiotic prophylaxis with amoxicillin plus clavulanic acid (2.4 g); antithrombotic prophylaxis was given for 30 days (low molecular weight heparin, 0.4 mL subcutaneously once a day); and octreotide prophylaxis was also given (0.1 mg subcutaneously for 3 times/24 hour for 5 days after the intervention.

Patients were divided into two groups according to the following: (a) the presence or absence of post-operative complications;(b) the median value of urine amylase levels measured on POD1 (140.00 U/L);(c) the median value of serum amylase levels measured on POD1 (75.50 U/L);and (d) the median value of amylase levels in drains measured on POD1 (971.50 U/L).

Statistical analysis was performed using SPSS version 17.0 (Chicago, IL, USA). Continuous data are presented as the means ± SD and were compared with an unpaired Student’s test and linear regression analysis. Categorical variables were reported as frequencies (%) and were compared with the Chi-square test or Fisher’s exact test. Relative risks with 95% confidence intervals (CIs) are presented. Univariate Cox proportional hazard regression models were used to determine the prognostic value of variables. The sensitivity and specificity of the urine amylase level were determined by ROC analysis. Correlations among variables were determined by Pearson’s correlation test. *p*<0.05 was considered significant.

The study was approved by the ethical committee of University Clinical Center Tuzla No: 02-09/2-39-20.

## RESULTS

Fifty-two patients who underwent Whipple resection were enrolled. There were 29 males (55.80%; mean age 64.97±9.49) and 23 females (54.20%; mean age 66.61±8.27). Indications for Whipple resection were as follows: ductal carcinoma 31 (59.60%), carcinoma of the papilla Vateri 6 (11.5%), adenocarcinoma of the papilla Vateri 4 (7.70%), endocrine tumors 3 (5.80%), and serous cystic neoplasms 8 (15.40%).

Out of 52 patients who underwent Whipple resection, 27 (51.90%) of them had post-operative complications. Types of complications after Whipple resection are shown in Table 1. The most frequent post-operative complication was delayed gastric emptying, which was observed in 23 (44.20%) patients. As the most severe complication, POPF grade C (POPF grade C) was observed in 9.60% of cases.

**TABLE 1:**
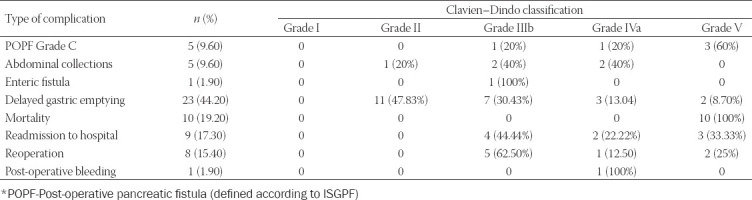
Types of post-operative complications after 52 Whipple resections

The serum C-reactive protein (CRP) level on POD1 was significantly higher than that before Whipple resection in both analyzed groups, in patients without and with complications (125.95 ± 12.58 vs. 53.05 ± 8.54 mg/L, *p*<0.000; and 132.76 ± 13.38 vs. 61.59 ± 7.12 mg/L, *p*<0.000, respectively).

**FIGURE 1:**
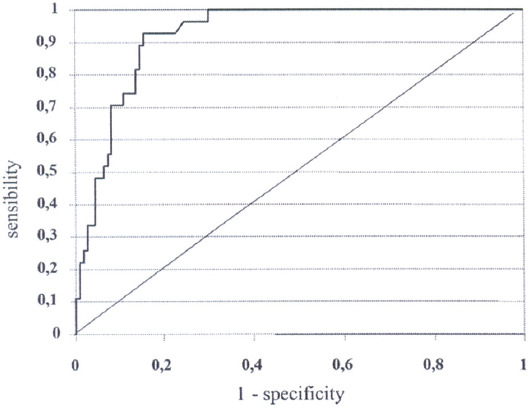
ROC curve based on urine amylase levels on POD1 (area under the curve 0.918; 95% CI: 0.894-0.942).

The urine amylase level on POD1 in patients with post-operative complications after Whipple resection was significantly higher than that in patients without complications (198.89 ± 28.41 vs. 67.04 ±2.67 IU/L, *p*<0.000) (Table 2). Considering the sensitivity and specificity of the urine amylase level on POD1, an area under the ROC curve of 0.918 was obtained (*p*<0.001, 95% CI: 0.894-0.942) (Figure 1). Patients were divided into two groups according to the median value of urine amylase levels on POD1 (140.00 U/L). Patients with urine amylase levels ≥140.00 U/L had significantly higher risks of POPF grade C (RR:20.26; 95% CI: 1.18-347.07; *p*=0.038), readmission to hospital (RR: 6.61; 95% CI: 1.53-28.58; *p*=0.011), reoperation (RR: 5.67; 95% CI: 1.27-25.27; *p*=0.023), and mortality (RR:17.00; 95% CI: 2.33-123.80; *p*=0.005) than patients with urine amylase levels <140.00 U/L(Table 3).

**TABLE 2:**
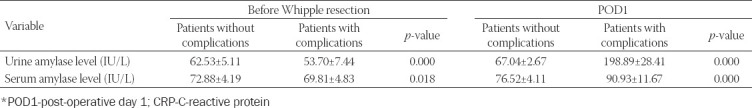
Comparison of urine and serum amylase levels before Whipple resection and on POD1 in patients with versus without complications

**TABLE 3:**
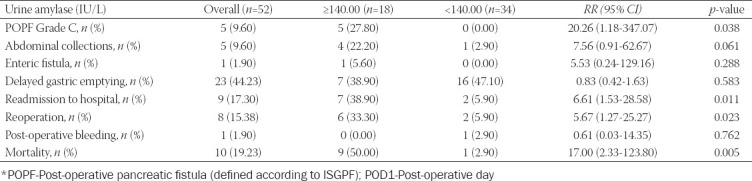
Risk stratification of patients after Whipple resection based on elevated urine amylase level (greater than the median value 140.00 IU/L) on POD1

The serum amylase level on POD1 in patients with post-operative complications after Whipple resection was significantly higher than that in patients without complications (90.93 ± 11.67 vs. 76.52 ± 4.11 IU/L, *p*<0.000) (Table 2). Patients were divided into two groups according to the median values of serum amylase levels on POD1 (75.50 U/L). Patients with serum amylase levels ≥75.50 U/L had significantly higher risks of POPF grade C (RR: 9.87; 95% CI: 1.19-81.19; *p*=0.033), delayed gastric emptying (RR: 1.89; 95% CI: 1.08-3.34; *p*= 0.026), readmission to hospital (RR:19.73; 95% CI: 2.69-144.42; *p*=0.003), reoperation (RR: 17.27; 95% CI: 2.32-128.56; p=0.005), and mortality (RR: 49.88; 95% CI: 3.11-800.97; p=0.006) than patients with serum amylase levels <75.50 U/L (Table 4).

**TABLE 4:**
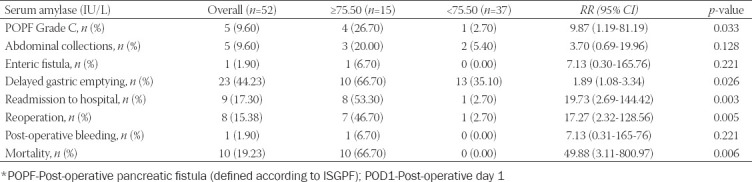
Risk stratification of patients after Whipple resection based on elevated serum amylase level (greater than the median value 75.50 IU/L) on POD1

The amylase level in drains on POD1 in patients with post-operative complications after Whipple resection was significantly higher than that in patients without complications (5052.22±2871.68 vs. 664.12±341.45 IU/L, *p*<0.000). Patients were divided into two groups according to the median value of amylase levels in drains on POD1 (971.50 U/L). Patients with amylase levels in drains ≥971.50 U/L had significantly higher risks of POPF grade C (RR: 31.43; 95% CI: 1.85-532.79; *p*=0.017), abdominal collections (RR: 31.43; 95% CI: 1.85-532.79; *p*=0.017), delayed gastric emptying (RR: 3.27; 95% CI: 1.94-5.53; *p*=0.000), readmission to hospital (RR: 54.29; 3.37-873.26; *p*=0.005), reoperation (RR: 48.57; 95% CI: 2.99-788.05; *p*=0.006), and mortality (RR: 60.00; 95% CI: 3.76-958.51; *p*=0.004) than patients with amylase level in drains <971.50 U/L (Table 5).

**TABLE 5:**
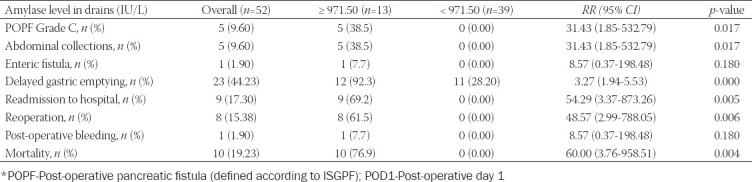
Risk stratification of patients after Whipple resection based on elevated amylase level in drains (greater than the median value 971.50 IU/L) on POD1

Urine amylase levels measured on POD1 displayed strong and significant positive correlations with serum amylase levels (*r*=0.92, *p*=0.001), amylase level in drains (*r*= 0.86, *p*=0.002), and CRP (*r*=0.95, *p*=0.000).

## DISCUSSION

Whipple resection still remains one of the most sophisticated procedures in abdominal surgery. It is the standard procedure in cases of benign or malignant pancreatic head or periampullary area. Post-operative mortality has decreased to <3% within the past decades due to technical and medical advances [2]. However, in our study, mortality rate was 19.20%. It is most probably due to the small hospital volume or/and surgeon’s experience. Despite advances in surgical techniques and perioperative management within the past decades, post-operative morbidity remains high [1,2].Cameron et al. [2] reported delayed gastric emptying (23%) as the most frequent morbidity. In our study, the delayed gastric emptying (44.20%) was the most frequent post-operative complication as well. However, it was significantly higher, most probably due to the small hospital volume, or/and the surgeon’s experience. As the most severe complication, POPFs are present in 11-16% of cases [2,4,13,14]. In large volume and experienced pancreatic surgery centers, the incidence rate of POPF C is decreased to <5% [9,15]. However, in our small pancreatic surgery center with less experience, it was 9.60%. We found similar results in the case of reoperation and readmission rate.

In our study, we found that in patients with complications, serum CRP was significantly higher on POD1 than before resection. There is a large number of studies that have reported serum CRP as an excellent predictor of post-operative complications after pancreaticoduodenctomy, some of which were performed by Pecorelli et al. [16], Vilhav et al.[17], Ma et al.[18],and Qu et al.[19].

In the present study, the serum amylase level on POD1 in patients with post-operative complications after Whipple resection was significantly higher than that in patients without complications (90.93 ± 11.67 vs. 76.52 ± 4.11 IU/L, p<0.000). Patients with serum amylase levels ≥75.50 U/L had significantly higher risks of POPF Grade C (RR: 9.87; 95% CI: 1.19-81.19; p=0.033), delayed gastric emptying (RR: 1.89; 95% CI: 1.08-3.34; *p*= 0.026), readmission to hospital (RR:19.73; 95% CI: 2.69-144.42; *p*=0.003), reoperation (RR: 17.27; 95% CI: 2.32-128.56; *p*=0.005), and mortality (RR: 49.88; 95% CI: 3.11-800.97; *p*=0.006) than patients with serum amylase levels <75.50 U/L. Increased serum amylase levels have been proven to be a very good predictor of the development of pancreatic fistula after pancreaticoduodenctomy [20-24]. Our results are similar to these ones.

The amylase level in drains on POD1 in patients with post-operative complications after Whipple resection was significantly higher than that in patients without complications (5052.22±2871.68 vs. 664.12±341.45 U/L, *p*<0.000). Patients with amylase levels in drains ³971.50 U/L had significantly higher risks of POPF Grade C (RR: 31.43; 95% CI: 1.85-532.79; *p*=0.017), abdominal collections (RR: 31.43; 95% CI: 1.85-532.79; *p*=0.017), delayed gastric emptying (RR: 3.27; 95% CI: 1.94-5.53; *p*=0.000), readmission to hospital (RR: 54.29; 3.37-873.26; *p*=0.005), reoperation (RR: 48.57; 95% CI: 2.99-788.05; *p*=0.006), and mortality (RR: 60.00; 95% CI: 3.76-958.51; *p*=0.004) than patients with amylase levels in drains <971.50 U/L. Our results are consistent with similar results reported in several recent studies [9,25-28].

To the best of our knowledge, we are the first to report an independent association between urine amylase levels and post-operative complications after Whipple resection. Patients with urine amylase levels ≥140.00 U/L had significantly higher risks of POPF Grade C (RR:20.26; 95% CI: 1.18-347.07; *p*=0.038), readmission to hospital (RR: 6.61; 95% CI: 1.53-28.58; *p*=0.011), reoperation (RR: 5.67; 95% CI: 1.27-25.27; *p*=0.023), and mortality (RR:17.00; 95% CI: 2.33-123.80; *p*=0.005) than patients with urine amylase levels <140.00 U/L. Considering the sensitivity and specificity of the urine amylase level on POD1, an area under the ROC curve of 0.918 was obtained (*p*<0.001, 95% CI: 0.894-0.942).

Urine amylase levels measured on POD1 displayed strong and significant positive correlations with serum amylase levels (*r*=0.92, *p*=0.001), amylase levels in drains (*r*=0.86, *p*=0.002), and CRP (*r*=0.95, *p*=0.000).

Limitations of this study were as follows: (a) It was based on a relatively small cohort from a single center. Compared with large clinical trials, this analysis should not be regarded as statistically robust; a larger population of patients who underwent Whipple resection is necessary to generalize our urine amylase findings;(b) although we assessed urine amylase levels before and on POD1 after the intervention, serial measurements might be more useful for evaluating its prognostic value and estimating risks during the follow-up period.

## CONCLUSION

Urine amylase levels on POD1 may be used as an additional prognostic marker of post-operative complications after Whipple resection for short-term outcomes. Determination of urine amylase levels on POD1 and other predictors may help create better risk classification for patients who undergo Whipple resection.
